# Raman spectroscopic molecular fingerprinting of biomarkers for inflammatory bowel disease

**DOI:** 10.1002/ctm2.1345

**Published:** 2023-11-03

**Authors:** Emma Buchan, Jonathan James Stanley Rickard, Pola Goldberg Oppenheimer

**Affiliations:** ^1^ School of Chemical Engineering Advanced Nanomaterials Structures and Applications Laboratories, College of Engineering and Physical Sciences University of Birmingham Birmingham UK; ^2^ Department of Physics Cavendish Laboratory University of Cambridge Cambridge UK; ^3^ Biochemical Engineering Healthcare Technologies Institute Institute of Translational Medicine Birmingham UK

Dear Editor,

Inflammatory bowel diseases (IBDs) are increasingly difficult to diagnose and differentiate despite serological testing, endoscopic and histopathological assessments. No single biomarker exists to predict IBD in a timely manner. We have developed a spectral library of candidate IBD‐biomarkers, establishing characteristic molecular barcodes, through a combination of multiplex spectroscopic profiling simultaneously detecting a panel of identified biomarkers with an advanced artificial intelligence (AI) network and classifying patients according to disease state. This lays the platform for rapidly and non‐invasively detecting IBD and discriminating between the subtypes for timely diagnoses, biomarker discovery, patient stratification and further potential significant developments of diagnostic methodologies and therapeutic monitoring.

IBDs are chronic inflammatory conditions affecting ∼3 million individuals in Europe with the incidences continuing to rise worldwide,^[^ ^1^, ^2^ ^]^ affecting any part of the digestive tract, from mouth to anus, with inflammation penetrating deep into the layers of the intestinal wall.^[^ ^2^, ^3^ ^]^ These complex illnesses characterised by acute and chronic states, mean individuals experience periods of inflammatory flare‐up, remission, and relapse. Whilst there is no cure for IBD, management strategies include therapeutics, surgery, and lifestyle alterations to improve symptoms and quality of life. The complexity of interactions between host factors and the dynamic fluctuation of gut microbiota hinders the identification of consistent changes in microbial composition and thus, the identification of universal biomarkers for IBD prediction.^[^ ^4^ ^]^ A large proportion of patients with non‐specific abdominal pain often undergo unnecessary, painful endoscopy or colonoscopy, which are not suitable for routine IBD diagnosis. A pool of diagnostic and/or prognostic biomarkers would considerably reduce the need for invasive, nonspecific procedures and greatly improve early diagnosis, management, and interventions. While the challenges of accurate diagnosis and monitoring of IBD have created an urgent need for rapid detection of the indicative biochemical changes, currently no biomarkers under investigation (Supporting Information S1) or suitable point‐of‐care sensing techniques exist that can address such a critical need for the 21st century.

Herein, we introduce Raman spectroscopy for methodical profiling and classification of potential IBD‐indicative biomarkers, establishing unprecedented *biochemical ‘fingerprints’* as characteristic barcodes for current and emerging diagnostic and prognostic studies. We systematically evaluate spectra of multiple potential IBD‐biomarkers, compare these with fingerprints of saliva and tissue biopsies and investigate these to gain insight into the underlying IBD immune and inflammatory processes, identifying specific cytokines, which are either over‐or‐underproduced, providing important clues to the underpinning mechanism of disease and future therapeutic targets. The data is classified using our new artificial neural network algorithm, ‘SKiNET’, as a decision support tool (Supporting Information‐S2/Figure S1). The overall established spectroscopic workflow puts forward several potential molecular biomarkers, whilst demonstrating clinical applications including biomarker discovery and patient stratification, laying the platform for defining their functionality in the complex aetiology of IBD (Figure 1b,c).

**FIGURE 1:**
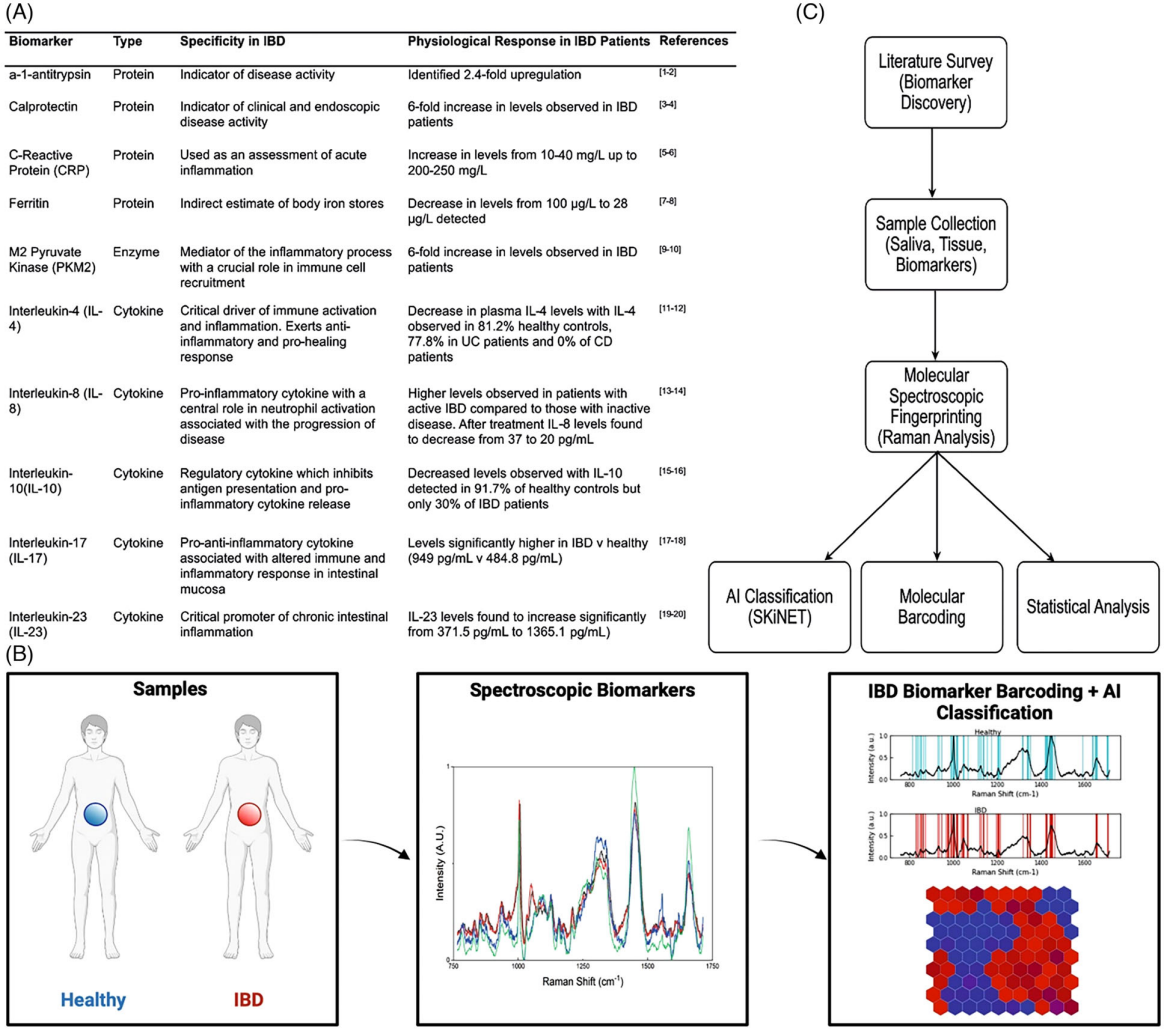
The established spectroscopic workflow, from quantitative spectral acquisition, through multivariate analyses and onto fingerprint barcoding and artificial intelligence (AI) classification including the (a) overview and rationale behind the selected candidate biomarkers, describing their specificity in inflammatory bowel diseases (IBD) alongside the recognised physiological response in IBD patients. Cytokines, known to play an active role in inflammation were chosen for their ability to contribute to the inflammatory status of the intestine, CRP as an overall indicator of inflammation, M2‐Pyruvate Kinase as a mediator of the inflammatory process, calprotectin allowing evaluation of gut inflammation and α−1‐antitrypsin as an indicator of disease progression. Each biomarker identified in the literature has a known physiological response in IBD patients with a statistically significant increase or decrease observed such as a 6‐fold increase in PKM2 levels, 2.4‐fold upregulation in α−1‐antitrypsin and a decrease in IL‐8 levels from 37 to 20 pg/ml. In this workflow (b) biofluid samples were obtained from patients, and analysed to identify unique spectroscopic signatures using a comprehensive multiplex Raman analysis with subsequent barcoding and advanced self‐optimising Kohonen index NETwork (SKiNET) machine learning for validation and classification. Raman's capability of multiplex detection, rapidly identifying several markers of disease simultaneously,^[^ ^5^, ^6^ ^]^ renders it particularly suitable for timely IBD diagnostics in contrast to the current laboratory‐based tests, which are limited to single target analyte detection and rarely provide the needed rapid multiplex biomarker detection, whilst early‐stage diagnostics necessitates monitoring the levels of at least 3−20+ biomarkers simultaneously. (c) The overall workflow describing the individual components and their correlation within the developed Raman profiling and spectroscopic fingerprinting towards candidate IBD biomarkers discovery (Supporting Information S5).

A panel of potential IBD‐biomarkers including cytokines IL‐4, IL‐8, IL‐10, IL‐12, IL‐17, IL‐23, Ferritin, M2‐pyruvate kinase (PKM2), Calprotectin, CRP and *α*−1‐antitrypsin (Figure 2), chosen based on their specificity for IBD and known physiological response in patients (Figure 1a), was assessed via hybrid Raman spectroscopy (Figure 2) and subsequently, classified via optimised AI algorithm with inherent self‐organised map discriminant index (SOMDI) spectroscopic fingerprints analysis of saliva and tissue biopsies (Figure 3c–f), assigning the dominant spectral signatures (Table S1/Figure S1) and yielding the characteristic barcodes (Figure 4).

**FIGURE 2:**
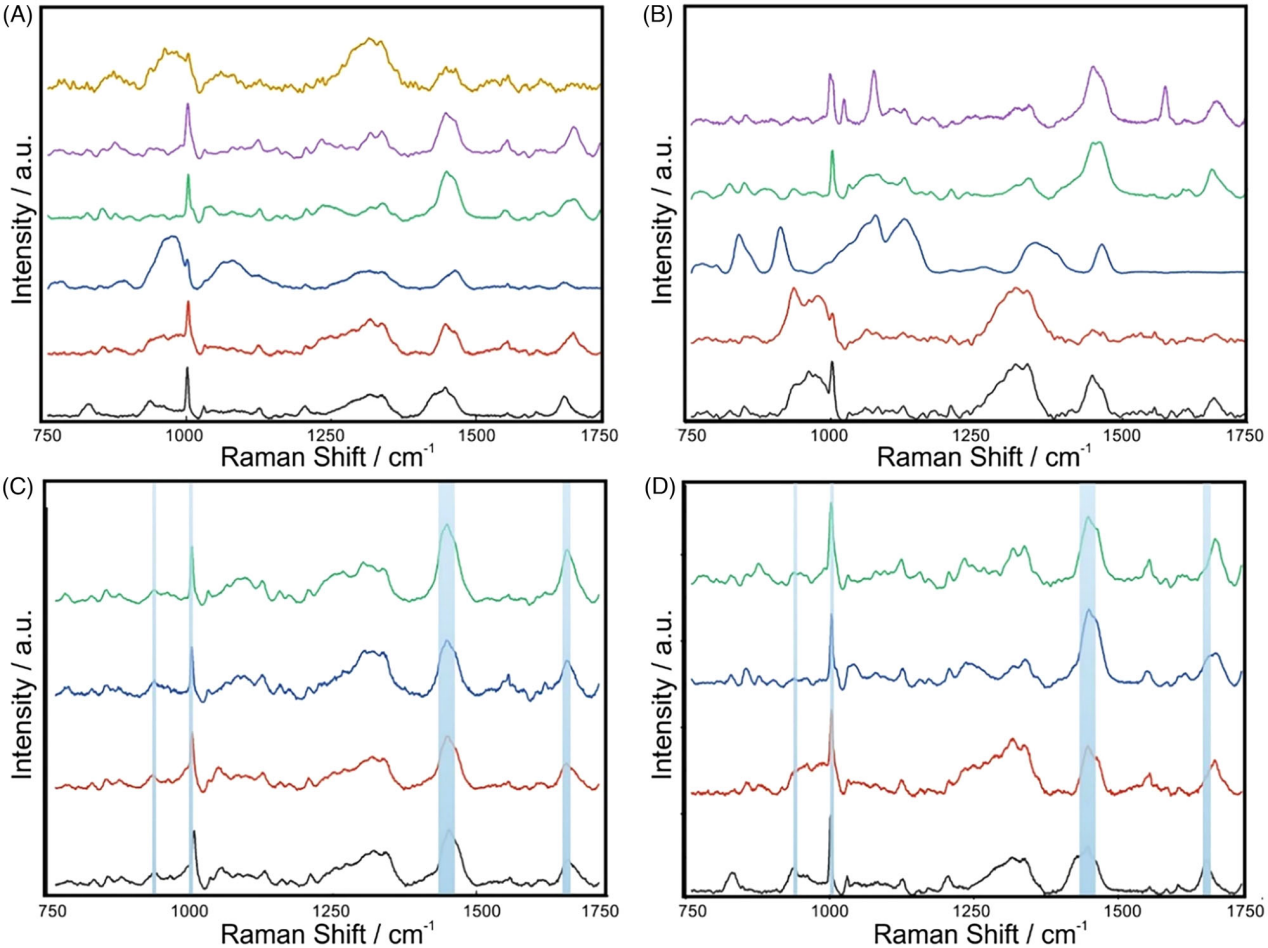
Average Raman spectra of candidate inflammatory bowel diseases (IBD) biomarkers including (a) cytokine profiling of IL‐4 (black), IL‐8 (red), IL‐10 (blue), IL‐12 (green), IL‐17 (purple), IL‐23 (light brown) and (b) a‐1‐antitrypsin (black), calprotectin (red), C‐reactive protein (blue), ferritin (green) and M2‐Pyruvate kinase (purple). Mean representative spectra of (c) saliva of healthy (black) and IBD (red) and biopsy tissue of healthy (blue) and IBD (green) patients and of the identified IBD biomarkers (d) IL‐4, IL‐8, IL‐12, and IL‐17 highlighting the peaks of interest at 936, 1003, 1340, 1445 and 1656 cm^−1^. The detected primary peaks at 936 cm^−1^ (protein), 1003 cm^−1^ (phenylalanine), 1340 cm^−1^ (C‐H deformation in proteins) 1445 cm^−1^ (δ(CH_2_ deformation of proteins) and 1656 cm^−1^ (Amide‐I) provide the fingerprints of both IBD saliva and tissue biopsy. Notably, the peak at 936 cm^−1^ exhibit a decreased intensity in the IL‐4 due to the C‐C stretch of the amino acids in the protein backbone.

**FIGURE 3:**
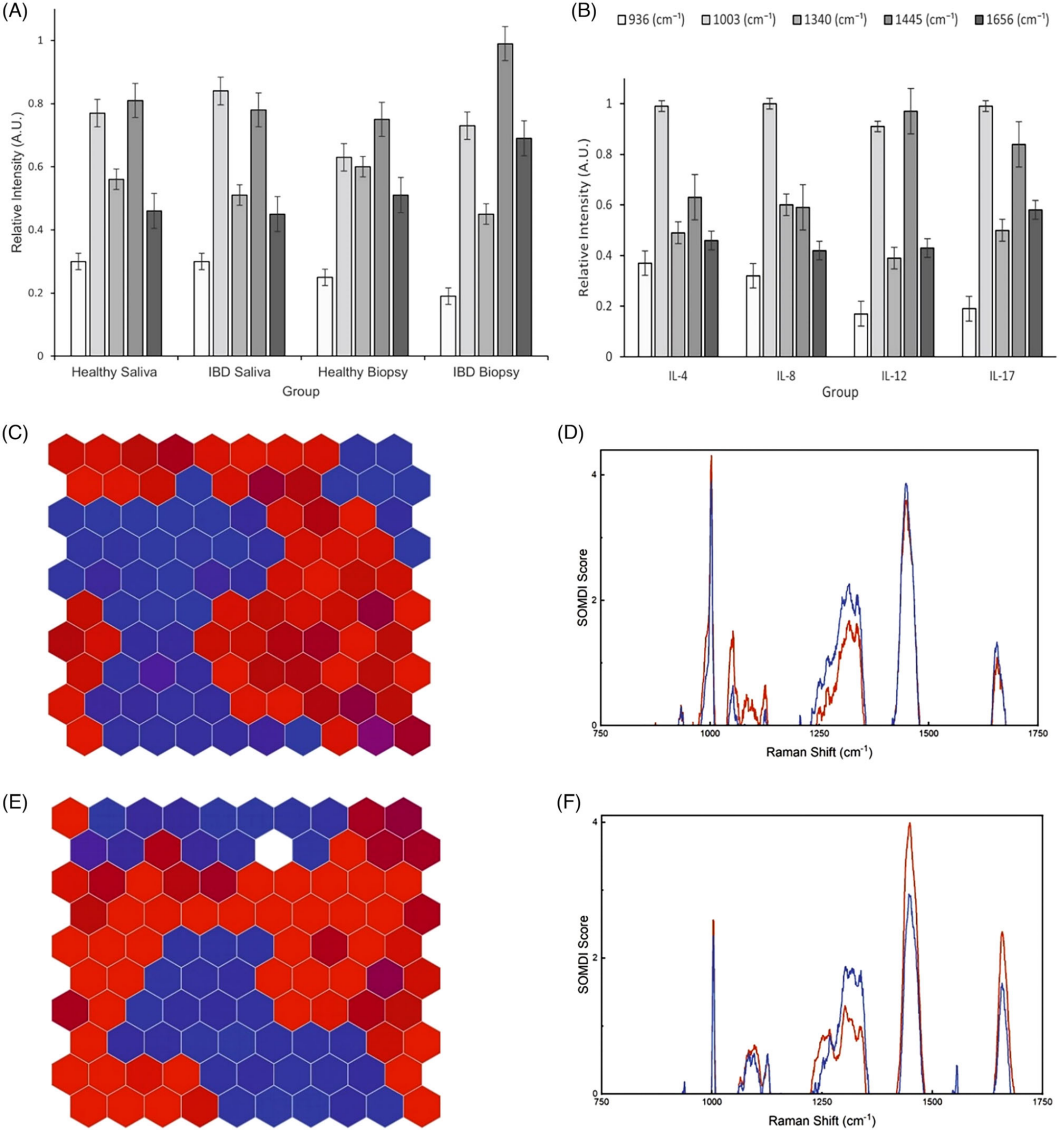
Histograms of the relative intensities of the dominant Raman peaks identified in (a) healthy and inflammatory bowel diseases (IBD) saliva and tissue biopsies and (b) potential IBD biomarkers (Error bars indicate the standard error). Spectral data is normalised using standard‐normal‐variate (SNV) normalisation prior to machine learning to enable enhanced and accurate comparison and analysis via subtraction of each spectrum from its own mean and a subsequent division by the standard deviation. Representative self‐organising map (SOM) classification according to the disease state of IBD (red) and healthy (blue) of (c) saliva and (e) tissue biopsy with the corresponding self‐organising map discriminant index (SOMDI) extracted features (d and f) from SOMs, highlighting the most influential Raman bands responsible for the clustering observed in SOM (c and e), classifying the patient samples according to diseased state with an accuracy of 88.5% and 86.8%, respectively. Comparison of saliva and tissue biopsies reveals distinct spectral changes with dominant bands at 936 cm^−1^ (protein), 1003 cm^−1^ (phenylalanine), 1110 cm^−1^ (C‐C bond), 1340 cm^−1^ (C‐H deformation), 1445 cm^−1^ (δCH_2_ deformation of proteins/lipids) and 1656 cm^−1^ (Amide‐I) identified via SOMs with significant spectral features extracted via SOMDI, distinguishing between control and IBD cohorts. The change in intensity of the 936 cm^−1^ mode in IBD saliva and tissue biopsy for IL‐8, IL‐12 and IL‐17 is associated with the C‐C stretching in amino acids with the increased number of hydrogen bonds leading to its weakening.

**FIGURE 4:**
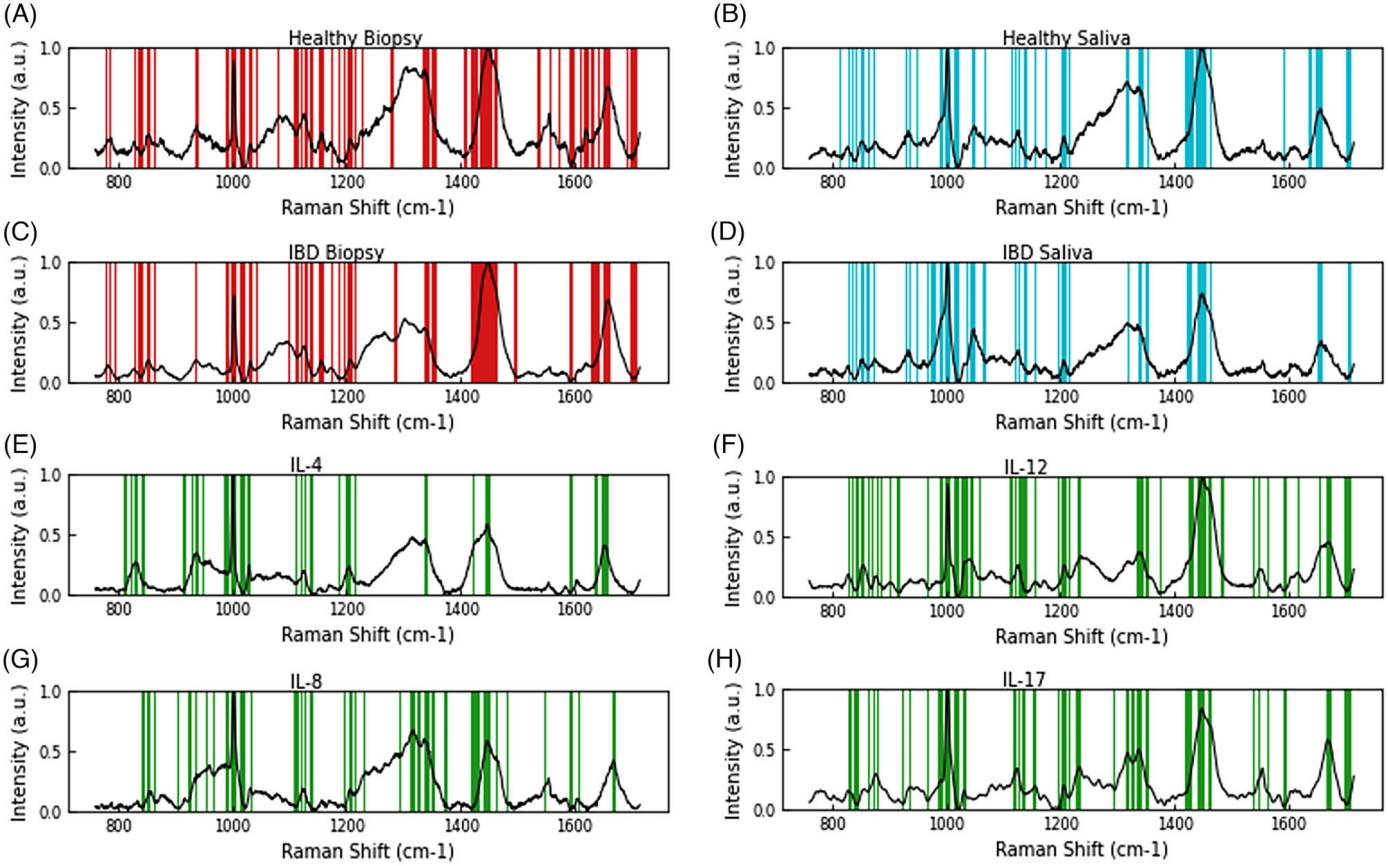
Barcodes derived from (a) and (c) tissue biopsy, (b) and (d) saliva and potential inflammatory bowel diseases (IBD) biomarkers (e) IL‐4, (f) IL‐12 (g) IL‐8 and (h) IL‐17, highlighting the dominant, statistically significant peaks. Overall, the hybrid Raman spectroscopy, combined with the advanced artificial intelligence (AI) algorithm of SKiNET‐SOMDI supervised machine learning, used to establish the complete linked workflow from projection, through classification to the development of molecular fingerprinting, introduces the spectroscopic approach for profiling and classification of IBD‐indicative biomarkers, enabling the assessment of biomarkers for inflammatory bowel disease. The derived spectroscopic molecular barcodes (a–h) combined with the identified biochemical changes, closely matching notable biomarker characteristics, establish a group of potential first‐line screening of IBD‐biomarkers.

Cytokines IL‐4, IL‐8, IL‐12 and IL‐17 exhibited specific Raman bands, providing identification and discrimination of IBD (Figure 2a), where the detected peaks yield fingerprints of saliva and tissue biopsies (Figure 2c,d/Supporting Information S3). We have subsequently compared the relative peak intensities of cytokines with those in IBD saliva and biopsies, identified via SKiNET and SOMDI, selecting those with the greatest influence on the classifier (Figure 3/Table S2). This yielded the detection of several Raman key‐bands, which were characteristic in patients with IBD including the 936 cm^−1^ (C‐C stretching‐amino acids, protein backbone), 1003 cm^−1^ (Phenylalanine), 1340 cm^−1^ (C‐H protein deformation), 1445 cm^−1^ (*δ*(CΗ_2_) deformation of proteins and lipids) and 1656 cm^−1^ (C = O proteins stretch, Amide‐I). We further discovered considerably downregulated 936, 1340 cm^−1^ and upregulated 1003, 1445 and 1656 cm^−1^ spectral signature in IBD patients (Supporting Information S3).

Levels of IL‐4 were decreased for healthy individuals when compared with their IBD counterparts and increased for IL‐8, IL‐12 and IL‐17 as reflected via the SOMDI‐identified significant Raman shifts (Figure 3d–f). Given their widely accepted role in IBD, via detecting the changes discriminating between diseased and healthy individuals, we highlight the significance of these markers in the pathogenesis of IBD (Figure 3/Supporting Information S3). The decreased intensity of IL‐4 at 936 cm^−1^ correlates with the known changes in pro‐inflammatory cytokines, which mediate the interaction between immune and non‐immune cells, previously shown to contribute to the inflammatory status of the intestine, where IBD‐related IL‐4‐mediated downregulated activation has been impaired.^[^ ^7^ ^]^ The ∼27% upregulation in the 1003 cm^−1^, detected for each of the cytokines, is indicative of an increased inflammatory response due to a greater influx of inflammatory cells in the diseased state. In IBD, neutrophils are known to be important cellular mediators with the IL‐8 being a powerful chemoattractant found in increased quantities in mucosa. Marked increased levels of IL‐8 at 1003 and 1656 cm^−1^ are indicative of protein conformational changes, suggesting IL‐8 is a significant indicator of disease with a potential to monitor mucosal healing and response to therapeutics. The statistically significant detected (p*** < 0.0001) increases of these bands combined with the decrease at 1445 cm^−1^ are indicative of IL‐12, known to play a key role in the activation and regulation of multiple cytotoxic immune cells including macrophages, natural killers and T cells,^[^ ^8^, ^9^ ^]^ highlighting its importance in following the pathogenesis and progression of IBD. A further ability of IL‐17, exerting a strong inflammatory response, to act as a multiplexed IBD biomarker is established with evident increases at 1003 and 1656 cm^−1^.^[^ ^10^ ^]^ The remaining examined biomarkers yielded spectral fingerprints, which did not match those identified in IBD saliva or tissue, despite acknowledged roles in inflammation and IBD pathogenesis. Overall, the detected cytokine changes via Raman profiling are central to the identification of IBD (*see ‘Extended Discussion’*: Supporting Information S3).

Comparison of saliva and tissue biopsies via the SkiNET enabled rapid data classification, revealing distinct spectral changes with dominant SOMDI‐identified bands, distinguishing between control and IBD cohorts (Figure 3), with the changes in protein and amino acid conformation and structure yielding the main differences in the spectral fingerprints (Supplementary Information‐S3/TablesS3‐S4). The spectroscopic profiling with the corresponding peak assignments and the dominant changes determined to be statistically significant for detecting IBD‐biomarkers (p*** < 0.0001), yielded the biomolecular barcodes (Figure 4).

Previously, Smith and Pence focussed on the classification of mucosal healing versus active inflammation and IBD characterisation. Our results correlating with these studies, via the ability to identify differing intensities within IBD and healthy cohorts, highlight the significance of IL‐4 in the pathogenesis of disease given its widely accepted role in IBD along with its diagnostic value and the potential to monitor mucosal healing and drug delivery. The detected changes further emphasise the importance of IL‐12 in the pathogenesis and progression of IBD and of the IL‐17′s ability to act as a multiplex IBD‐biomarker.

Overall, herein, Raman spectroscopy has been successfully employed as a rapid, non‐invasive technique for multiplexed profiling, establishing unique molecular barcodes for candidate biomarkers from saliva and tissue biopsies, including a range of inflammatory cytokines, yielding an efficient combination of specific potential IBD key indicators. Our approach combines the ability to not only classify saliva and tissue as healthy versus disease but also, use the obtained molecular fingerprints to identify the IBD‐associated changes within both clinical sample types and correlate these to IBD‐indicative biomarkers via the derived barcodes. These real‐time in‐vivo spectroscopic measurements in IBD patients could enable establishing insights into biological pathways underlying the associated pathophysiology and conceivably, allow tracking the passage and dosage of current and emerging pharmacological therapeutics. This also lays the platform for defining their functionality in the complex IBD aetiology along with cementing Raman spectroscopy as a useful technique for future biomarker discovery in various detrimental diseases with many ramifications.

## CONFLICT OF INTEREST.STATEMENT

The authors declare no conflict of interest.

## Supporting information



Supporting Information

## Data Availability

The data supporting the findings of this study are available in the ‘*Supporting Information’*.
