# Supplementary material for: Raman spectroscopic molecular fingerprinting of biomarkers for inflammatory bowel disease
Source: Clin Transl Med. 2023 Nov 3;13(11):e1345. doi: 10.1002/ctm2.1345 (PMC10623648; doi:10.1002/ctm2.1345)
Supplement: Supplementary file 1 — Supporting Information [file CTM2-13-e1345-s001.docx]

**- Supplementary Information -**

**Raman Scattered Spectroscopic Molecular Fingerprinting of Biomarkers for Inflammatory Bowel Disease**

*Emma Buchan^1^, Jonathan James Stanley Rickard^2^* and *Pola Goldberg Oppenheimer^1,3, *^*

Raman provides unique biomolecular spectral fingerprints of target analytes with rapid analytical response, enabling non-destructive, label-free, quantitative analysis of composition and structure with an inherently straightforward detection and no complex sample preparation, thus rendering itself as a powerful technique, which can be used to *in-situ* measure various biomarkers, yielding quantitative information about their concentration, highly-useful for monitoring disease progression and response to treatments. The availability of portable instruments makes it particularly attractive for point-of-care detection.

**S1. Potential IBD Indicative Biomarkers Under Investigation**

Collection of biofluids, *e.g.,* blood / urine, is a standard procedure in routine clinical practice and hence is ideal for a point-of-care or bedside setting. Biofluid derived biomarkers have many useful applications in healthcare including disease prevention and detection, determination of individual’s risk and disease monitoring. [1-2] Currently, however, there are no validated blood biomarkers to accurately and reliably predict IBD. The complexity of interactions between host factors and the dynamic fluctuation of the gut microbiota during IBD hinders the identification of consistent changes in microbial composition, and thus the universal biomarkers for disease prediction. [3] Hence a large proportion of patients with non-specific abdominal pain often undergo unnecessary painful endoscopy or colonoscopy to rule out IBD. [4] Whilst effective, these are invasive and painful, and thus less suitable for routine use for timely and rapid IBD diagnosis. Not only are these procedures invasive but also a confirmatory IBD diagnosis requires further clinical examinations, imaging, and pathological investigations, which can be often inconclusive. A pool of diagnostic and/or prognostic biomarkers of IBD would thus, considerably reduce or eliminate the need for invasive or nonspecific procedures and greatly improve early-stage diagnosis, management, and therapeutic interventions for IBDs. The ability to determine the type, severity and patient response to therapeutics has long been a priority of clinical researchers.

Although demonstrating correlation with IBD severity and holding potential diagnostic, prognostic and stratification value, biomarkers currently under investigation, are undetectable unless analysis is carried out in specialised laboratories.

Potential IBD indicative biomarkers currently under investigation include C-reactive protein (CRP), faecal calprotectin, anti-*Saccharomyces cerevisiae* antibodies (ASCA), anti-neutrophil cytoplasmic antibodies (ANCA) and serum amyloid A [5-9]. Cytokines, proteins produced by immune cells, are also known to play an active role in inflammation, and thus are additional candidate biomarkers in IBD. [10] Idiopathic IBDs typically arise in clinically immunocompetent individuals with cytokine-driven inflammation of the gastrointestinal tract giving rise to the distinctive signs and symptoms in individuals. [11] CD is most often associated with increased levels of IL-12/IL-23 and IFN-γ/IL-17, whereas UC is associated with excess IL-13 production. [12] Research studying the pathogenesis of IBD suggests that IBD arises due to a dysfunctional interaction between the mucosal immune system and the bacterial microflora of the gastrointestinal (GI) tract, and thus cytokines are well known to play a key-role in controlling intestinal inflammation and the associated diseases. [13] Cytokine testing therefore has the potential to support the IBD diagnosis due to its low cost and lack of invasiveness, compared to routine IBD diagnostics.

While demonstrating correlation with IBD severity and holding potential diagnostic, prognostic and stratification value, these potential biomarkers are undetectable unless analysis is carried out in specialised laboratories, using enzyme-linked immunosorbent assay (ELISA), chemiluminescent assay, high performance liquid chromatography (HPLC) or mass spectrometry. Although reliable, these techniques have time-to-results in the range of days, require complex equipment and trained personnel and do not meet the need for detection of ultra-low levels of target analytes in complex biological samples using rapid and direct readouts. ELISAs are relatively cost-effective but, despite advancements, suffer from poor stability, not rapid enough, require expensive labels for each biomarker and result in unacceptable rates of false negatives and false positives. [14]

[1]. Soubières AA. Emerging role of novel biomarkers in the diagnosis of inflammatory bowel disease. World J Gastrointest Pharmacol Ther. 2016, 7(1):41.

[2]. Wang A, Wang C, Tu M, Wong D. Oral Biofluid Biomarker Research: Current Status and Emerging Frontiers. Diagnostics. 2016 17, 6, 45.

[3]. Khan I, Ullah N, Zha L, Bai Y, Khan A, Zhao T, et al. Alteration of Gut Microbiota in Inflammatory Bowel Disease (IBD): Cause or Consequence? IBD Treatment Targeting the Gut Microbiome. Pathogens. 2019 13, 8(3):126.

[4]. Tefas C, Mărginean R, Toma V, Petrushev B, Fischer P, Tanțău M, et al. Surface-enhanced Raman scattering for the diagnosis of ulcerative colitis: will it change the rules of the game? Anal Bioanal Chem. 2021 7, 413(3):827–38.

[5]. Wakai M, Hayashi R, Tanaka S, Naito T, Kumada J, Nomura M, et al. Serum amyloid A is a better predictive biomarker of mucosal healing than C-reactive protein in ulcerative colitis in clinical remission. BMC Gastroenterol. 2020 3.20(1):85.

[6]. ROOZENDAAL C, KALLENBERG CGM. Are anti-neutrophil cytoplasmic antibodies (ANCA) clinically useful in inflammatory bowel disease (IBD)? Clin Exp Immunol. 2001, 24, 116(2):206-13.

[7]. Israeli E. Anti-Saccharomyces cerevisiae and antineutrophil cytoplasmic antibodies as predictors of inflammatory bowel disease. Gut. 2005 1;54(9):1232.

[8]. Sherwood R, Walsham N. Fecal calprotectin in inflammatory bowel disease. Clin Exp Gastroenterol. 2016 Jan;21.

[9]. Vermeire S, van Assche G, Rutgeerts P. C-Reactive Protein as a Marker for Inflammatory Bowel Disease. Inflamm Bowel Dis. 2004 Sep;10(5):661–5.

[10]. Rogler G, Andus T. Cytokines in Inflammatory Bowel Disease. World J Surg. 1998 1, 22(4):382-9.

[11]. Strober W, Fuss I, Mannon P. The fundamental basis of inflammatory bowel disease. Journal of Clinical Investigation. 2007 1, 117, 514.

[12]. Guan Q, Zhang J. Recent Advances: The Imbalance of Cytokines in the Pathogenesis of Inflammatory Bowel Disease. Mediators Inflamm. 2017, 2017,1-8.

[13]. Fava F. Intestinal microbiota in inflammatory bowel disease: Friend of foe? World J Gastroenterol. 2011, 17(5):557.

**S2. Self-Optimising Kohonen Index Network (SKiNET) Algorithm**

The acquired data is classified using our new artificial neural network algorithm, self-optimising Kohonen index network (SKiNET) as a decision support tool, based on self-organising map (SOM) with a classification *via* the self-organising map discriminant index (SOMDI).

Through inspection of key differences between neuron weights and class weight vectors, the algorithm enables identification of the key spectral changes. These allow the identification of the types of data a given neuron activates, which are then used to inspect the weights across all neurons and extract prominent features belonging to each class by finding the weights that contribute most to a particular class. The peaks in SOMDI subsequently correspond to cm^-1^ and modes that contribute most to the clustering observed in the SOM. Training parameters used for the SOM include grid size, learning rate and optimal number of epochs and the separation of classes reveals the characteristic differences due to the classification of certain neurons. This enables a clear basis for differentiation *via* the characteristic weight vectors to be derived in SOMDI.

Inspired by the visual cortex in the brain, SOMs are trained for the neighbouring neurons to activate according to similar inputs, in this case Raman spectra. Each neuron has a weight vector with length equal to the number of variables in a spectrum. Through exposing the network to training samples over a number of iterations, the weights are gradually adjusted to be similar to the input data, so that each neuron only activates on a given spectral signature. The result is a projection of hyperspectral data into 2D space, that can be shown as visible clustering according to sample / disease type and state. SKiNET inherently employs SOMDI, which appends a set of label vectors to each neuron and allows us to study the most prominent features that cause the activation of a particular neuron to a class label. Subsequently, a supervised learning step is introduced to optimise the network, and the class label associated with each neuron used to quickly identify new data presented to the SOM, allowing for diagnostics. Raw component spectra from chosen candidate molecules are fitted to SOMDI for a particular state, constituting a physically realistic fit, as our Raman spectra represent a mixed state of positive contributions from constituent components.

Our AI is based on SOMs with SKiNET as a framework for multivariate analysis that simultaneously provides (i) dimensionality reduction, (ii) feature extraction and (iii) multiclass classification (**Fig. S1**), where SKiNET performs visual separation to identify the underlying chemical differences between classes, providing accurate classification for simultaneously rich-information and high-classification specificity. SOMs provide visually intuitive 2D-clustering (according to disease / healthy *etc*. state) of high-dimensional data Raman spectra, that are otherwise difficult to interpret for large sample and measurement numbers. SKiNET incorporates supervised learning to additionally provide accurate classification, which could then be used to make diagnostic predictions. Whilst previous SOMs are usually an unsupervised method, our optimised SKiNET incorporates supervised learning to additionally provide accurate classification, which could then be used to make diagnostic predictions. Finally, a form of feature extraction (SOMDI) allows to understand which spectral features (=biochemical changes) are responsible for the clustering seen in SOMs.

When Raman spectroscopy combined with SKiNET is applied to investigate whether the identified biomarkers reflect the IBD, it distinguishes disease from a healthy control group, showing this to be because of similar chemical changes detected.

This algorithm enables a clear separation of the data from the different tissue or biofluid classes arranged as SOM, trained on spectra from these classes. Neurons (hexagons) are coloured according to the modal class they activate, from the training set of Raman spectra. Neurons that have no majority class or activate none of the training data are shown in white. Coloured circles within each neuron represent spectra from the training data that have been activated for that neuron. To aid visualisation, circles are forced to not overlap in space using the D3force library, providing an alternative mechanism to display sample frequency and class overlap for each neuron. For each class, there is a clearly defined block of neurons, with many of these activating only a single tissue type. An approximately even distribution in the number of neurons required to identify each class is observed. The SOMDI provides a representation of weights associated with neurons that identify a particular class. A higher SOMDI intensity indicates a greater importance of particular inverse centimetres along the axis of a spectrum. This, despite the level of overlap or noise in the original data, enables well defined peaks to be resolved, which are either more prominent or unique to each class. Automated classification of Raman spectra and assignment to a particular biomarker, tissue type or disease state is perhaps the most important step for the translation of Raman based diagnostic techniques to real world, clinical applications.

**Figure S1.** Illustration of data analysis pipeline using SKiNET. Spectra measured from Raman **(a)** of saliva or tissue are grouped according to class **(b)**. A 20% partition of the data is randomly selected and reserved as test data **(c)**. The remaining 80% is input into SKiNET, which directly provides dimensionality reduction (SOM), self-organising map discriminant index (SOMDI) feature extraction and classification **(d)**. SKiNET is optimised on the training data using cross validation and adjusting the available parameters (number of neurons, initial learning rate and number of training steps) to maximise the classification accuracy on the training data. Finally, the optimised model is shown the previously unused test data and asked to classify each spectrum as either IBD, healthy or IBD biomarker.

**Table S1.** Characteristic assignments for the identified dominant Raman peaks. [1-5]

| **Raman Shift / cm^-1^** | **Assignment** |
| --- | --- |
| 759 | Tryptophan ring breathing mode; Proteins |
| 828 | DNA phosphate groups |
| 875 | C-C symmetric stretching; Proteins and lipids |
| 877 | Choline stretching group; Phosphatidylcholine |
| 936 | C-C stretch amino acids; Protein Backbone |
| 1003 | Protein marker, Phenylalanine ring breathing mode, ρ(C-C); Phenylalanine |
| 1110 | C-C vibration of the gauche-bonded chain |
| 1051 | C-O and C-N stretch |
| 1080 | (ν(C-C) of lipids); PO_2_ group in DNA and lipids |
| 1128 | Trans C-C stretching; backbone of lipid, protein, and carbohydrate |
| 1201-1207 | CH_2_ wagging vibrations (glycine, proline, tyrosine, and phenylalanine |
| 1230-1285 | Amide III; Protein |
| 1270 | C-H deformation; Lipids |
| 1305 | CH_2_ twisting; Phospholipids, adenine, myoglobin |
| 1337 | CH_2_/CH_3_ wagging and twisting (proteins, nucleic acids), nucleic acid bases (υ(C-H)) |
| 1340 | C-H deformation; Protein |
| 1368 | ω(CH_2_), δ(CH), υ4-υ(Pyr ¼ ring); tryptophan, guanine, thymine, myoglobin (Haem core) |
| 1445 | Shoulder (δ(CH_2_) deformation of proteins and lipids |
| 1447 | CH_2_ scissoring (lipid and protein) |
| 1518 | υ(C=C) |
| 1620-1680 | Amide I band |
| 1622 | C=C stretching mode of tyrosine and tryptophan |
| 1643 | C=C stretching modes of lipids and the protein Amide I |
| 1656 | C=C stretching in lipids, C=O stetch in proteins |
| 1658 | (υ(C=O)) |

[1]. Tefas C, Mărginean R, Toma V, Petrushev B, Fischer P, Tanțău M, et al. Surface-enhanced Raman scattering for the diagnosis of ulcerative colitis: will it change the rules of the game? Anal Bioanal Chem. 2021, 7;413(3):827-38.

[2]. Morasso C, Truffi M, Vanna R, Albasini S, Mazzucchelli S, Colombo F, et al. Raman Analysis Reveals Biochemical Differences in Plasma of Crohn’s Disease Patients. J Crohns Colitis. 2020, 7;14(11):1572-80.

[3]. Pence IJ, Beaulieu DB, Horst SN, Bi X, Herline AJ, Schwartz DA, et al. Clinical characterization of in vivo inflammatory bowel disease with Raman spectroscopy. Biomed Opt Express. 2017, 1, 8(2):524.

[4]. Smith SCL, Banbury C et al. Raman spectroscopy accurately differentiates mucosal healing from non-healing and biochemical changes following biological therapy in inflammatory bowel disease. PLoS One. 2021;16, 1-16.

[5]. Pence I, Mahadevan-Jansen A. Clinical instrumentation and applications of Raman spectroscopy. Chem Soc Rev. 2016, 45, 1958.

**Table S2.** Normalised Raman intensity of main statistically significant and dominant spectral peaks identified.

| **Marker** | **Raman Shift / cm^-1^** | | | |  |
| --- | --- | --- | --- | --- | --- |
|  | **936** | **1003** | **1340** | **1445** | **1656** |
| **Healthy Saliva** | 0.29 | 0.77 | 0.56 | 0.81 | 0.46 |
| **IBD Saliva** | 0.28 | 0.84 | 0.51 | 0.78 | 0.45 |
| **Healthy Biopsy** | 0.25 | 0.63 | 0.60 | 0.75 | 0.51 |
| **IBD Biopsy** | 0.19 | 0.73 | 0.45 | 0.99 | 0.69 |
| **IL-4** | 0.37 | 0.99 | 0.49 | 0.63 | 0.45 |
| **IL-8** | 0.28 | 1.00 | 0.60 | 0.59 | 0.42 |
| **IL-12** | 0.17 | 0.91 | 0.39 | 0.97 | 0.43 |
| **IL-17** | 0.19 | 0.99 | 0.50 | 0.84 | 0.58 |

**Table S3**. Classification performance of tissue biopsy compared to healthy controls including sensitivity, specificity, positive predictive values (PPV) and negative predictive values (NPV).

| **Comparison** | **Sensitivity** | **Specificity** | **PPV** | **NPV** | **Accuracy** |
| --- | --- | --- | --- | --- | --- |
| Healthy v. IBD | 83.9 | 84.6 | 87.6 | 88.2 | 82.5 |
| Healthy v. UC | 94.7 | 90.4 | 90.0 | 95.0 | 90.0 |
| Healthy v. CD | 94.4 | 86.3 | 85 | 95 | 90.2 |

**Table S4**. Classification performance of saliva compared to healthy controls.

| **Comparison** | **Sensitivity (%)** | **Specificity (%)** | **PPV** | **NPV** | **Accuracy** |
| --- | --- | --- | --- | --- | --- |
| Healthy v. IBD | 87.5 | 89 | 89 | 87.3 | 88.3 |
| Healthy v. UC | 91.6 | 88.1 | 88.5 | 88.5 | 80.1 |
| Healthy v. CD | 87.3 | 87.7 | 86.1 | 88.9 | 80.3 |

**Figure S2.** Raman spectral data of non-normalised molecular fingerprints of **(a)** saliva and **(b)** tissue biopsy (blue=healthy, red=IBD). The identified Raman spectral fingerprints obtained from non-normalised data align with the observed SNV normalised data, with the only differences observed in the scaling of the data.

**S3. Spectroscopic Data Analyses and Interpretation Details – Extended Discussion**

A trio of characteristic bands at 821, 851, 924cm^-1^ were identified for PKM2, emerging as a significant mediator of inflammatory processes with serum levels 6-fold higher in IBD *versus* healthy patients, [1] posing PKM2 as a putative IBD-biomarker (**Fig.2b/S3**). Prior research by Czub *et* al. identified increased fecal PKM2 levels 100% of active CD patients and 94.3% of active UC patients with associated enzyme reactivity higher in all IBD patients than those healthy controls. Here we identified PKM2 at increased levels in both saliva and tissue at each of the three characteristic Raman bands, thus indicating its potential as an identifier of IBD in both tissue and saliva as opposed to its current use in faecal samples. In addition, whilst CRP exhibited increased intensities at 841, 912, 1076 and 1126cm^-1^ (*p****<0.0001), this marker alone was found to be non-specific due to the elevation in systemic inflammatory disease other than those identified within the intestinal tract, for example in cancers of the body or infections, CRP levels can also be significantly elevated. However, when combined with other potential markers of IBD, such as PKM2, IL-4, IL-8, IL-12 and IL-23 CRP provides invaluable insight into the level of inflammation in the gut. Increased levels of CRP for example, can help differentiate mucosal active disease from quiescent IBD as well as acting as a predictor as to the need for colectomy through the reflection of severe ongoing and uncontrolled inflammation of the gut. [2]

Notably, the peak at 936cm^-1^ exhibits a decreased intensity in the IL-4 compared with IL-8, IL-12, and IL-17 due to the C-C stretch of the amino acids in the protein backbone, where the vibrational frequency depends on the local chemical environment including hydrogen bonds and steric hindrance and represents the local conformation of the peptide bond predominantly influenced by the neighbouring amino acid residues. The 1340cm^-1^ is attributed to C-H deformation of proteins backbone/side chains, providing information on the presence of hydrogen bonds, electrostatic interactions and intermolecular forces. Since the position and intensity of this band can be influenced by presence of ligands, cofactors, or changes in the native environment, analysing these provides important underpinning insights on the structure and dynamics of the proteins. Herein, the rise in the C-H deformation band indicates an increase in either number or strength of C-H bonds in each of the interleukin molecules, and a change in the protein secondary structure due to the diseased state of the patient.

The peak at 1656cm^-1^ is associated with the amide-I band of the C=O stretching of backbone conformation. Given that amide-I is the most intense absorption band in proteins and its exact position is determined by the backbone confirmation and the pattern of hydrogen bonding, its increase is indicative of these changes.

**Figure S3.** Representative SOM clustering and classification according to biomarker type of **(a)** all the studied biomarkers, **(c)** CRP, calprotectin, α-1-antitrypsin, ferritin and PKM2 and **(e)** cytokine; IL-4, IL-8, IL-10, IL-12, IL-17 and IL-23 with the corresponding SOMDI extracted features from SOM **(b, d and f)** classifying biomarker subtype with an accuracy of 98.2%.

We have successfully detected and identified several Raman key-bands, which were characteristic in patients with IBD including the 936cm^-1^ (C-C stretching of amino acids, protein backbone), 1003cm^-1^ (Phenylalanine), 1340cm^-1^ (C-H deformation, Protein), 1445cm^-1^ (Shoulder (δ(CΗ2) deformation of proteins and lipids and 1656cm^-1^ (C=O stretch in proteins, Amide I). In IBD disease state, we also found that the intensity of the spectral peaks at 936 and 1340cm^-1^ to be considerably downregulated, whilst the bands at 1003, 1445 and 1656cm^-1^ were upregulated. These results are consistent with study Morasso *et* al. [4] where the authors identified similar assignments to these spectral changes via comparison of dried plasma *ex-vivo* samples using Raman spectroscopy and analysed using principal component analysis followed by linear discriminant analysis.

The fact that the same set of peaks are visible in both the healthy and diseased sample types would thus indicate that their biochemical composition is similar, which is consistent with our general understanding of inflammation. Inflammation does not introduce new metabolites into the system but instead, leads to an overproduction or overuse of the existing metabolites. Therefore, it is expected to see the same molecular changes as identified *via* intensity changes of the peaks in the same region in both tissue types as was evidenced in our study, supporting that these are due to the inflammatory response, where the variation in peak intensities between the two types are thus due to the detected differences in the concentration of the biomolecules. Furthermore, the significant increase in intensity at 1003cm^-1^ is an additional indication of inflammation due to IBD, since in previous studies, this peak has been associated with higher levels of phenylalanine, due to the immune activation and inflammation. Smith *et* al. observed a significant reduction in intensity due to mucosal healing and Addis et al. recognised a similar effect, following biological therapy. [5] An additional finding relating to the peak changes at 1656cm^-1^ (Amide I) could indicate conformational changes induced by factors evolving from the inflammatory state. Although, it is possible that factors stemming from age, dehydration or radiological conditions, could also be responsible for the changes detected, these do not affect the data reported in our study as the recruited subjects were of a highly similar age, with no known health conditions thus predominantly, the biochemical changes detected are indicative of the inflammatory response associated with IBD.

Our study subsequently, has proceeded to identify which biomarkers associated with IBD could be responsible for the intensity changes found between healthy and IBD patient samples within these regions. Changes in response to IBD, have shown downregulation at 936cm^-1^, (representative of C-C stretching associated with amino acids such as glutamine and arginine which influence the progression of IBD in individuals) with the main statically significant differences arising from amino acid composition (*p****<0.001). Our results of decreased intensity at 936cm^-1^ in IBD patients is further consistent with previous studies, where changes in pro-inflammatory cytokines, which mediate interaction between immune cells and non-immune cells, have been shown [6-9] to contribute to the inflammatory status of the intestine. In ulcerative colitis, the T-helper 2 response consists of IL-4 and IL-13. IL-4 is an important contra inflammatory cytokine, which limits monocyte and macrophage activation. IL-4-mediated downregulation of activation has been shown by Ruckert *et* al. to be impaired in IBD. [10] Hoving *et* al. have also indicated that mice deficient in IL-4, IL-13 and IL-4 receptor-alpha on all cells develop an exacerbated IBD phenotype. [11] A further study by Xiong *et* al. identified the effects of IL-4 and IL-10 gene therapy on TNBS-induced murine colitis, shown to significantly inhibiting the TNBS-induced colon tissue damage and disease activity index as well as a marked block in expression of IFN-γ and TNF-α, highlighting the significance of monitoring IL-4 levels in IBD patients as well as the potential of IL-4 as a first line of defence in the treatment of IBD .[9]

Furthermore, we have detected an upregulation of the band at 1003cm^-1^ attributed to the phenylalanine changes indicative of an increased inflammatory response. In IBD, neutrophils are known to be important cellular mediators with the IL-8 being a powerful neutrophil chemoattractant found in increased quantities in the mucosa. Grimm *et* al., by isolating macrophages and monocytes from intestinal resections and detecting IL-8 by *in-situ* hybridisation, found that in the inflamed bowel, IL-8 was detected in both macrophages and neutrophils however, not detected in uninflamed mucosa and the IL-8 expression was significantly more common by macrophages from IBD affected mucosa than in their healthy counterparts. [12] These results support that the detected increased levels of IL-8 with marked intensity increase at 1003cm^-1^ combined with an increase in the Amide I band at 1656cm^-1^ are indicative of protein confirmational changes, suggesting that IL-8 can act as a significant indicator of disease. Additionally, previous study by Billiet *et* al. identifying the correlation of primary IFX therapy response with lower serum IL-8 levels, further suggests the ability of cytokines to predict the response to different therapies used in patients with IBD. [6]

Additionally, the detected increase of statistically significant (*p****<0.0001) bands at 1003 and 1656cm^-1^ combined with the decrease at 1445cm^-1^ are indicative of the IL-12, playing a key-role in the activation and regulation of multiple cytotoxic immune cells including macrophages, natural killer cells and T cells, [6,13] produced predominantly by macrophages in response to bacteria and their products. IL-12 is rarely detected in normal intestinal mucosa and therefore, acts as an appealing potential marker of IBD. Recently, IL-12, in combination with other interleukins such as IL-23, has been emerging as a target molecule for treatment in IBD patients, for example, the use of Ustekinumab, an anti-IL-12/23p40 antibody has been approved for CD. Almradi *et* al. have also indicated that in early stages of IBD, IL-12 is the dominant p40-containing cytokine, driving inflammation in response to intestinal barrier disruption. [14] Similarly, IL-17 is known to exert a strong proinflammatory response. Its secretion has been reported to be limited to T lymphocytes with major indications of it being a strong mediator of inflammatory response in various tissues [15] as well as enhancing the proinflammatory response induced by IL-1β and TNF-α. Previous studies have shown that IL-17 expression has been detected in inflamed mucosa of active UC and CD patients *via* immunohistochemical techniques and serum ELISA. [16] Levels of IL-17 have been also previously found to increase significantly from 371.5 pg/mL to 1365.1 pg/mL in patients with IBD. [17]

Building upon the above studies correlating with our results, *via* the ability to identify differing intensities within IBD and healthy individuals, we firstly highlight the significance of IL-4 in the pathogenesis of disease given its widely accepted role in IBD as well as the diagnostic value of IL-8 as a biomarker with its potential to monitor mucosal healing and potential monitoring of patients in responses to therapeutics. The detected changes further highlight the importance of IL-12 in the pathogenesis and progression of IBD and of the IL-17 ability to act as a multiplexed IBD biomarker. Overall, real-time, rapid *in-vivo* spectroscopic measurements in IBD patients will enable establishing insights into biological pathways underlying the associated pathophysiology and could conceivably allow tracking the passage and dosage of current and emerging pharmacological therapeutics.

[1]. Czub E, Herzig KH, Szaflarska-Popawska A, Kiehne K, Socha P, Woś H, et al. Fecal pyruvate kinase: a potential new marker for intestinal inflammation in children with inflammatory bowel disease**.** Scand J Gastroenterology**.** 2007 10, 42(10): 1147-50.

[2] Vermeire S, Assche GA, Rutgeerts P, C-reactive protein as a marker for inflammatory bowel diease. Inflammatory Bowel Disease. 2004 9, 10(5):661-5.

[3]. Tefas C, Tanțău M. Clinical applications of raman spectroscopy in inflammatory bowel diseases. A review. Journal of Gastrointestinal and Liver Diseases. 2018, 27(4):433-8.

[4] Marasso C, truffe M, Vanna R, et al. Raman analysis reveals biochemical differences in plasma of Crohn’s disease patients. Journal of Crohn’s and Colitis, 2020, 1572-1580.

[5] Addis J, Mohammed N, Rotimi O, et al. Raman spectroscopy of endoscopic colonic biopsies from patients with ulcerative colitis to identify mucosal inflammation and healing. Biomedical Optics Express, 2016, 7(5).

[6]. Billiet T, Cleynen I, Ballet V, Claes K, Princen F, Singh S, et al. Evolution of cytokines and inflammatory biomarkers during infliximab induction therapy and the impact of inflammatory burden on primary response in patients with Crohn’s disease. Scand J Gastroenterol. 2017 3, 52(10):1086-92.

[7] West G, Matsuura T, Levine A, et al. Interleukin 4 in inflammatory bowel disease and mucosal immune reactivity, 1996, 110(6):1683-1695.

[8] Jansson J, Willing B, Lucio M, et al. Metabolomics reveals metabolomic biomarkers of Crohn’s Disease. PLoS One, 2009, 4(7):e6386.

[9] Xiong J, Lin Y, Bi L, et al. Effects of Interleukin-4 pr Interleukin-10 gene therapy on trinitrobenzenesulfonic acid-induced murine colitis, BMC Gastroenterology, 2013, 13 (165).

[10] Rückert Y, Schnidler U, Heinig T, et al. IL-4 Signalling mechanisms in inflammatory bowel disease mononuclear phagocytes, Inflamm Bowel Dis, 1996, 2(4):244-52.

[11] Hoving J, Keeton R, Höft M, et al. IL-4 receptor-alpha signalling intestinal epithelail cells, smooth muscle cells, and macrophages plays a redundant role in Oxazolone colitis. Mediators Inflamm, 2020, 32410852.

[12] Grimm M, Elsbury S, Pavli P, Doe W. Interleukin 8: cells of origin in inflammatory bowel disease, Gut, 1996, 38(1), pp.90-98.

[13] Greving C, Towne J. A role for IL-12 in IBD after all? Immunity, 2019, 51(2), pp. 209-211.

[14] Almradi A, Hanzel J, Sedano R, et al. Clinicla trials of IL-12/IL-23 inhibitors in inflammatory bowel disease. Clinical Trials BioDrugs, 2020, 34(6):713-721.

[15] Fujino S, Andoh A, Bamba S, et al. Increased expression of interleukin 17 in inflammatory bowel disease. Gut, 2003, 52(1):65-70.

[16] Lucaciu L, Ilies M, Vesa S, et al. Serum interleukin (IL)-23 and IL-17 profile in inflammatory bowel disease (IBD) patients could differentiate between severe and non-severe disease. J Pers Med, 2021, 11(11): 1130.

[17] Fujino S. Increased expression of interleukin 17 in inflammatory bowel disease. Gut. 2003, 1, 52(1):65-70.

**S4. Mass Spectrometry**

**Figure S4.** Mass spectrometry of the total ion chromatographs for **(a)** healthy and **(b)** unhealthy-IBD saliva, indicating the relative abundancies of all ions in each mass spectrum as highlighted in the corresponding time stamps.

Mass spectrometry was used to analyse both healthy and IBD saliva samples. Relative abundance of the majority of detected ions varied between the samples with the primary observed proteins and their corresponding coverage, as summarised in **Table S5**.

The majority of observed proteins varied significantly in their coverage between healthy and IBD samples particularly in those proteins involved in inflammation and the inflammatory cascade. Of a particular importance was the identification of IL-4, IL-8 and IL-17 receptors which were identified in IBD saliva with only IL-8 detected in healthy saliva. Also of note were the differing levels of S100-A9 and S100-A8 with both found at significantly higher levels in IBD saliva. These proteins are both known to have a role in inflammation with involvement in inducing secretion of pro-inflammatory cytokines including TNF-α and IL-1β, with these mediators understood to exaggerate and sustain inflammatory conditions. Increasing levels of S100A8 and A9 has also been shown to significantly increase protein release of Il-6 and IL-8. [1] In addition, there was a significant decrease in the levels of both alpha-amylase and mucin-5 in IBD patient sample compared with healthy indicative of differing salivary secretion rates between the groups. Pyruvate kinase was also identified in IBD patient saliva but was notably absent in healthy patient saliva, thus indicative of elevated levels in patients with IBD. Fecal pyruvate kinase is readily applied in the diagnosis of disease however, its presence in saliva observed with both Raman spectroscopy and mass spectrometry indicates its ability to act as a non-invasive salivary biomarker of IBD.

**Table S5.** Mass spectrometry protein assignment and coverage for healthy and IBD saliva samples.

| **Protein** | **Coverage (%)** | | **Molecular Weight (kDa)** |
| --- | --- | --- | --- |
|  | **IBD** | **Healthy** |  |
| S100-A9 | 36 | 20 | 13.2 |
| S100-A8 | 32 | 18 | 10.8 |
| Annexin A1 | 18 | 65 | 38.7 |
| Lipocalin-1 | 6 | - | 19.2 |
| Interleukin-8 | 4 | 1 | 8.4 |
| Matrix Metalloproteinase-9 | 1 | 1 | 78.4 |
| Pyruvate Kinase | 3 | - | 57.9 |
| Interleukin-17 | 2 | - | 35.0 |
| Alpha-amylase | 47 | 71 | 57.7 |
| Mucin-5 | 4 | 14 | 596 |
| Cornulin | 7 | 42 | 53.5 |
| Interleukin-1 receptor | 11 | 8 | 6.19 |
| Trefoil factor-3 | 12 | - | 10.2 |
| Interleukin-4 receptor | 2 | - | 14.9 |

[1] Crowe LAN, McLean M, Kitson SM, et al. S100A8 & S100A9: Alarmin mediated inflammation in tendinopathy. Nature Scientific Reports, 9, 2019.

**S5. Materials and Methods**

**Sample Collection and Preparation.** Saliva was collected from 101 participants (51 IBD and 50 healthy) at Queen Elizabeth Hospital Birmingham, UK. All healthy volunteers had no previous health issues or known conditions. 5 mL of unstimulated saliva was collected from each participant in a 50 mL Falcon tube (Thermo Fisher) *via* the passive drool method. Subsequently, 5 µL of raw saliva was pipetted onto an aluminium slide and air dried in an airtight container for 30 minutes. Colon tissue biopsies were collected from 44 participants (32 IBD and 12 healthy) (Ethics Refs. ERN_22-0290 and 19/SW/0010) into specimen containers (Thermo Fisher) containing 2 mL PBS and analysed immediately. All candidate markers were purchased directly from Sigma-Aldrich and Miltenyi Biotec Cologne and subsequently diluted in PBS (pH 8) to a working concentration of 1μg/mL. 5 μL of each was then pipetted onto an aluminium foil covered glass slide and dried in an airtight container for 30 minutes.

**Raman Spectroscopy.** Raman spectra were acquired using a Renishaw *InVia* Qontor confocal Raman microscope equipped with a microscope Leica DMLM and 785 nm laser (Renishaw PLC) with a spectral resolution of 0.3cm^-1^. Laser light was focussed using a x50 objective lens with a laser power of 5 mW. Spectral maps were acquired over an area of 50x50 μm^2^ in the fingerprint region of 700-1700 cm^-1^ with a 5 μm step size, 10 accumulations and 1s exposure time per spectrum. Overall, 100 spectra per sample were collected and used for data processing and analyses.

**Data Acquisition and Analyses.** The data was collected using WiRE 5.1 and further employed for the polynomial background subtraction and the removal of cosmic rays. Normalisation was done using the standard normal variate (SNV) (Python 3.7), which was used to derive the multi-chemical barcoding of the data. SNV is applied to each data point in the data set, whereby, the mean of all data points within the given dataset is subtracted from each individual data point thus centring the data around zero. Each centred data point is then divided by the standard deviation, scaling the data. Savitzky-Golay filter was applied to calculate the second derivative of each spectrum. Smoothing window was set to 21 with a polynomial order of 2. Maximum peak heights with absolute values over 40% were assigned a value of 1 with values below 40% assigned a value of 0. Values were overlaid on the averaged spectra with reference to the main peaks identified for the barcode generation. Statistical analysis was performed using a one-tailed students *t*-test, peaks identified as having a *p*<0.001 were determined as statistically significant.

**Data Processing and Multivariate Analysis.** Multi-variate analysis was performed using the self-optimising Kohonen index network based on self-organising maps (SOMs) with the accompanying Raman Toolkit web interface to build SOM models using training data and perform predictions against test data. SKiNET models were optimised by performing 10-fold cross validation on the training data, and tuning the number of neurons, initial learning rate and number of training steps. The final model used a 10x10 grid of neurons, 38,200 training steps (4 epochs of the data), with an initial learning rate of 0.1. The optimised model was subsequently used to classify the previously unused test data. Classification using the test data were repeated ten times from separate SOM initialisations. To achieve higher accuracy the SOM size, learning rate and number of epochs was empirically tested, with classification accuracy determined using a 10-fold cross validation. Stability of the model was further verified by running a repeat initialisation of the classification four times.
